# Primary care providers’ perceived barriers to obesity treatment and opportunities for improvement: A mixed methods study

**DOI:** 10.1371/journal.pone.0284474

**Published:** 2023-04-18

**Authors:** Lauren Oshman, Amal Othman, Wendy Furst, Michele Heisler, Andrew Kraftson, Yousra Zouani, Cheryl Hershey, Tsai-Chin Cho, Timothy Guetterman, Gretchen Piatt, Dina H. Griauzde

**Affiliations:** 1 Department of Family Medicine, University of Michigan Medical School, Ann Arbor, Michigan, United States of America; 2 Department of Internal Medicine, University of Michigan Medical School, Ann Arbor, Michigan, United States of America; 3 VA Ann Arbor Healthcare System, Ann Arbor, Michigan, United States of America; 4 University of Michigan Institute for Healthcare Policy and Innovation, Ann Arbor, Michigan, United States of America; 5 College of Engineering, Wayne State University, Detroit, Michigan, United States of America; 6 Department of Learning Health Sciences, Ann Arbor, Michigan, United States of America; University of South Australia, AUSTRALIA

## Abstract

**Background:**

Primary care patients with obesity seldom receive effective weight management treatment in primary care settings. This study aims to understand PCPs’ perspectives on obesity treatment barriers and opportunities to overcome them.

**Study design:**

This is an explanatory sequential mixed methods study in which survey data was collected and used to inform subsequent qualitative interviews.

**Settings and participants:**

PCPs who provide care to adult patients in an academic medical center in the Midwestern US.

**Methodology:**

PCPs (n = 350) were invited by email to participate in an online survey. PCPs were subsequently invited to participate in semi-structured interviews to further explore survey domains.

**Analytic approach:**

Survey data were analyzed using descriptive statistics. Interviews were analyzed using directed content analysis.

**Results:**

Among 107 survey respondents, less than 10% (n = 8) used evidence-based guidelines to inform obesity treatment decisions. PCPs’ identified opportunities to improve obesity treatment including (1) education on local obesity treatment resources (n = 78, 73%), evidence-based dietary counseling strategies (n = 67, 63%), and effective self-help resources (n = 75, 70%) and (2) enhanced team-based care with support from clinic staff (n = 53, 46%), peers trained in obesity medicine (n = 47, 44%), and dietitians (n = 58, 54%). PCPs also desired increased reimbursement for obesity treatment. While 40% (n = 39) of survey respondents expressed interest in obesity medicine training and certification through the American Board of Obesity Medicine, qualitative interviewees felt that pursuing training would require dedicated time (i.e., reduced clinical effort) and financial support.

**Conclusions:**

Opportunities to improve obesity treatment in primary care settings include educational initiatives, use of team-based care models, and policy changes to incentivize obesity treatment. Primary care clinics or health systems should be encouraged to identify PCPs with specific interests in obesity medicine and support their training and certification through ABOM by reimbursing training costs and reducing clinical effort to allow for study and board examination.

## Introduction

In the United States, rates of obesity (defined body mass index [BMI] ≥30 kg/m^2^) continue to rise, with estimates suggesting that approximately half of the adult population will have obesity by 2030 [1]. Individuals with obesity face an increased risk of physical and mental health conditions, including cardiovascular disease, type 2 diabetes, fatty liver disease, osteoarthritis, and depression [2]. Fortunately, even modest weight loss (5%) is associated with improvements in cardiometabolic risk factors, including reduced glucose, insulin, and triglycerides levels [3–6]. Greater weight loss (≥ 10%) can support additional health benefits, including improved sleep apnea and management of type 2 diabetes with fewer or no medications [3, 5, 7, 8].

Primary care providers (PCPs) are encouraged to play key roles in helping patients with obesity to lose weight and prevent, control, and reverse obesity-related chronic conditions [9]. Specifically, clinical practice guidelines [5, 10] and policies [11] urge PCPs to (1) identify patients with obesity, (2) inform patients of obesity treatment options, and (3) support individuals’ choice of and engagement in an individualized treatment plan. Treatment options include primary care-based lifestyle counseling (i.e., structured nutrition and exercise counseling for 6–12 months) [12], anti-obesity medications, medical weight management programs (e.g., very low-energy meal replacement), and bariatric surgery [13, 14].

Despite PCPs’ opportunity to help patients with obesity achieve weight loss, many face challenges that hinder their ability to provide evidence-based obesity treatment. Primarily, PCPs lack the time [15], knowledge [16, 17], and training [15, 18, 19] to routinely and effectively develop individualized weight management treatment plans [20]. When PCPs do provide counseling for weight management, it often consists only of general advice on diet and physical activity [21–24] without specific, evidence-based treatment or follow-up plans [25–27]. Taken together, these barriers contribute to an under-utilization of effective obesity treatments, including intensive lifestyle counseling as well as under-utilization of anti-obesity medications and referrals to bariatric surgery [22, 28–30].

While prior studies have characterized barriers to obesity treatment among PCPs, little is known about PCPs’ current obesity treatment practice patterns [9, 22] and perceived opportunities to improve obesity treatment. One such opportunity may be through training in obesity medicine through the American Board of Obesity Medicine (ABOM) [31]. ABOM offers physicians across all medical specialties the opportunity to obtain specialized knowledge of and competency in obesity treatment through continuing medical education or fellowship training. The number of US physicians certified in obesity management as ABOM Diplomates increased from 587 in 2012 to 5242 in 2021 [32]. ABOM certification continues to rise and most (65%) ABOM Diplomates are PCPs. Yet, given the high and rising obesity prevalence, additional efforts may be needed to increase the number of PCPs trained in obesity medicine, engage other primary care team members in delivering effective obesity treatment, and enhance use of the full range of evidence-based obesity treatment.

The primary aim of this mixed methods study is to explore the perspectives PCPs in a large, academic health system on opportunities to improve obesity treatment, including training in obesity medicine. To understand and contextualize perceived opportunities for improvement, we first explored (1) PCPs’ current obesity treatment practice patterns, with a focus on understanding PCPs’ use of health system- and community-based programs, self-help resources (e.g., apps), and anti-obesity medications, and (2) PCPs’ perceived barriers to obesity treatment.

## Materials and methods

This is an explanatory sequential mixed method study; quantitative data was collected in the first phase and used to inform the development of a qualitative interview guide. Interviews with PCPs then provided additional context to further elucidate quantitative findings [33, 34]. This study was approved by the University of Michigan Institutional Review Board (HUM00191496).

### Participants and recruitment

This study was conducted in a single, large academic health system in the Midwestern US. Participants were primary care providers (PCPs) specialized in Family medicine (FM), Internal Medicine (IM) and Combined Internal Medicine & Pediatrics (Med-Peds) with an active clinical practice in 1 of 14 primary care clinics. PCPs (n = 350) were invited by email to participate in an online survey in September 2020. Prior to completing the survey, participants provided informed consent electronically. At the conclusion of the survey, respondents indicated their willingness to participate in a brief interview to discuss survey topics in greater depth. In Spring 2021, PCPs who indicated willingness to participate in an interview (n = 41) were contacted by email and invited to schedule a 30-minute interview; ten survey respondents agreed to participate in an interview. An additional email was sent to all PCPs (n = 350) to solicit additional interview participation.

### Quantitative data collection

#### Survey measures

We developed a survey to explore PCPs’ obesity treatment practice patterns, perceived barriers to obesity treatment, and perceived opportunities for improvement. The survey was developed by the study team and iteratively revised based on feedback provided by one obesity medicine specialist and 5 PCPs. Individual survey question items were informed by clinical expertise, prior literature [15, 35], and obesity treatment guidelines [36]. The survey items are shown in S1 Appendix and citations are provided for items adapted from prior literature. The key survey domains are summarized below.

*Provider characteristics*. Participants reported gender, specialty, number of years in practice (not including residency training), and number of half days per week devoted to clinical care.

*Obesity treatment experience and knowledge*. Participants were asked to estimate the percent of their total patient panel with obesity and the percent of clinical encounters during which they made specific weight loss recommendations (beyond general diet and physical activity statements). Participants were additionally asked to report their perceptions regarding minimum percent body weight loss necessary to achieve health benefits and the effectiveness of obesity treatment options.

*Current obesity treatment practice patterns and barriers*. Participants were asked to report the frequency with which they use specific obesity treatment resources (i.e., dietitians s, anti-obesity medications, bariatric surgery) on a 5-point Likert scale from “very often” to “never.” Participants were asked to report their level of agreement with reasons why they may not make weight loss treatment recommendations during a clinic visit with patients with obesity on a 5-point Likert scale from “strongly agree” to strongly disagree.”

*Opportunities for improvement*. Using items adapted from the Organization Readiness to Change Assessment [37, 38], participants were asked to indicate their level of agreement with statements about whether their primary colleagues and clinical leadership considers obesity treatment to be a priority and whether the health system offers adequate weight management resources. Participants were asked to indicate resources that could better help them support weight loss among patients with obesity (e.g., more support from clinical staff, clinical decision support tools). Additionally, we explored participants certification in obesity medicine or interest in obtaining certification in obesity medicine through the ABOM [39].

#### Quantitative analysis

We performed descriptive statistical analysis using Stata, version 15 (Stata-Corp). Survey questions assessing the extent of agreement used a 5-point Likert scale (strongly agree to strongly disagree). Survey questions assessing frequency of use of obesity treatments and referrals used a 4-point frequency scale of very often (weekly) to never. Specific survey items and response options are shown in S1 Appendix. We constructed dichotomous measures to indicate positive responses (e.g., strongly agree and agree) compared to neutral or negative responses (e.g., neither agree or disagree, disagree, and strongly disagree) for analyses.

### Qualitative data collection

We integrated key constructs and results from the initial quantitative phase into a 16-question semi-structured qualitative interview guide (S2 Appendix). Topics included the initiation of weight loss discussions, treatment recommendations and resources used, and what additional support providers want and need. One author (LO) performed cognitive testing with an obesity specialist and two primary care physician and iteratively revised the interview guide. One author (YZ) conducted interviews by Zoom videoconferencing or phone between May and August 2021. Interview participants provided verbal informed consent prior to interview participation. Interviews lasted approximately thirty minutes in duration and participants were compensated for their time with a $40 gift card.

#### Qualitative analysis

Following verbatim transcription of the recorded interviews, three authors (DG, LO, and YZ) coded the initial interviews and created a code book using directed content analysis, meaning that codes were created to reflect the main topics in the interview guide and to reflect the patterns and themes that emerged from the data [40]. Two authors (YZ, CH) independently coded the remaining interviews, resolving coding discrepancies using consensus conference.

Qualitative codes were mapped to survey domains, including current practice patterns, barriers to obesity treatment and referral, and potential opportunities to overcome barriers. Within each domain, codes were subsequently organized to reflect key themes, as detailed below. Qualitative analysis was performed using NVIVO 12 (QSR International).

### Integrated analysis

Integration occurred at two points. First, the quantitative results informed the development of the qualitative semi-structured interview tool. We then used a deductive analysis approach to integrate the qualitative themes into the quantitative findings. We report meta-inferences, or conclusions that are based on integration of quantitative and qualitative findings, with a weaving approach [41, 42].

## Results

A total of 107 (30.6%) invited PCPs completed the quantitative survey. The majority (73.1%) were female, over half (56.1%) specialized in Family Medicine, nearly half (48.6%) had been in practice for more than ten years, and over fifty percent (54.2%) devoted at least half of their time to clinical practice (Table 1).

**Table 1:** Demographic characteristics of survey and interview participants.

	Quantitative Survey Participants (n = 107)	Qualitative Interview Participants (n = 16)
Demographics	N (%)	N (%)
Female	76 (73.1)	11 (68.8)
Specialty		
Family Medicine	60 (56.1)	7 (43.8)
Internal Medicine	37 (34.6)	9 (56.3)
Med-Peds	10 (9.4)	0
Years in practice after residency		
<5	33 (30.8)	5 (31.3)
5–10	22 (20.6)	3 (18.8)
>10	52 (48.6)	8 (50)
Half-days/week in clinical practice		
1–2	20 (18.7)	4 (25)
3–4	28 (26.2)	2 (12.5)
5–6	40 (37.4)	5 (31.3)
7+	18 (16.8)	5 (31.3)

Among 107 survey respondents, 41 (38.3%) expressed interest in qualitative interview participation. There were no differences in demographic and training characteristics between participants who volunteered (n = 41) for interview participation compared to this who did not (n = 66). Of the 41 PCPs who expressed interest in interview participation, 10 completed a qualitative interview; an additional six PCPs who did not initially express interest were invited and agreed to participate for a total of 16 interviewees. There were no differences in demographic and training characteristics between participants who completed a qualitative interview (n = 16) compared to those who volunteered and did not participate (n = 31).

### Quantitative results

#### Current PCP obesity treatment practice patterns

On average, survey participants estimated that over half (56%) of their patients have obesity, but they discuss weight management treatment during less than one-third (27%) of clinical encounters with these patients. Less than half of survey respondents (n = 45, 42.1%) reported 5% weight loss as the threshold at which patients with obesity may achieve health benefit. Approximately one-third of the survey respondents reported 10% weight loss as necessary for achieving health benefit (n = 36, 33%) and a minority (n = 16, 15%) reported that the health benefits of weight loss depended on a patient’s baseline weight. Almost all survey participants (n = 99, 92.5%) reported using their clinical judgment to guide obesity treatment. Few reported using evidence-based guidelines, including the Endocrine Society Clinical Practice Guideline on Pharmacologic Management of Obesity (n = 8, 7.5%), Obesity Medicine Association Algorithm (n = 5, 4.7%), 2013 AHA/ACC/TOS Guidelines for the Management of Overweight and Obesity in Adults (n = 5, 4.7%), or AACE/ACA Algorithm for Medical Care of Patients with Obesity (n = 2, 1.95%).

#### Barriers in obesity treatment and referral

Key barriers to obesity treatment included the presence of more urgent health concerns (n = 95, 88.8%) and short visit durations with inadequate time to discuss weight management (n = 93, 86.9%). Additional barriers are shown in Table 2. Most commonly, PCPs referred patients with obesity to dietitians (n = 89, 83.2%), community programs such as WW^TM^ (formerly Weight Watchers^TM^) or Diabetes Prevention Programs (n = 81, 75.7%), and self-help resources (e.g., mobile health tools, books, websites) (n = 75, 70.1%). Less than one-third (n = 32, 29.9%) of PCPs reported prescribing anti-obesity medications often or very often to their patients with obesity. Fewer PCPs referred to sub-specialty weight management programs available in the health system, including a very low-calorie meal replacement program offered through endocrinology (n = 23, 21.5%), an intensive lifestyle change program offered through preventive cardiology (n = 20, 18.7%), a support group for emotional eating (n = 18. 16.8%), bariatric surgery (n = 20, 18.7%), or endoscopic bariatric therapy (e.g., balloon device, aspiration device, or endoscopic sleeve gastroplasty) (n = 14, 13.1%).

**Table 2:** Barriers to obesity treatment in primary care settings (N = 107).

Reasons for no weight loss recommendations during a clinic visit	N (%)^a^
Patient has other health concerns or conditions that are more urgent	95 (88.8)
Short visit duration with insufficient time to discuss weight management	93 (86.9)
Patient does not have insurance coverage for available weight loss resources/programs	59 (55.1)
Losing weight is not a priority or goal for the patient	50 (46.7)
Available weight loss strategies are not effective	49 (45.8)
Patient already knows what he/she/they need(s) to do to manage their weight	42 (39.3)
Not believe that the patient will take the necessary steps to lose weight	37 (34.6)
Patient does not tell me he/she/they want(s) to lose weight	29 (27.1)
Not confident in my ability to make specific weight loss/control recommendations	26 (24.3)
Patient does not have any weight-related health problems	25 (23.4)
Worried about offending patients by discussing their weight	20 (18.7)
Cannot bill for conversations about weight loss	8 (7.5)
Not my role as a physician to counsel on weight loss	5 (4.7)
Helping patients to lose weight is not a priority	1 (0.9)

^a^Results are reported as aggregate of those responding ‘agree’ or ‘strongly agree’ to 5-point Likert scale question ranging from “strongly agree” to “strongly disagree”.

#### Opportunities to improve obesity treatment

Almost all participants agreed or strongly agreed that their PCP colleagues consider obesity treatment to be a priority (n = 90, 84.1%) and a majority reported that their clinical leadership considers obesity treatment to be a priority (n = 67, 62.6%). Few participants perceived existing clinic resources as adequate to support obesity treatment (n = 14, 15%), and identified key opportunities for improvement. Specifically, participants requested information on health system resources for obesity treatment (n = 78, 72.9%), training on effective dietary counseling (n = 67, 62.6%) and education about effective self-help resources for patients with obesity (n = 75, 70.1%). About half of survey respondents reported a need for increased reimbursement for obesity management (n = 56, 52.3%) and health system innovations to support effective obesity treatment, including increased access to dietitians (n = 58, 54.2%) and peer support programs (n = 47, 43.9%). Approximately one-third desired electronic medical record interventions to support obesity treatment such as order sets to guide prescribing of obesity treatment options (n = 38, 35.5%). Additional results are shown in Table 3.

**Table 3:** Opportunities to improve obesity treatment (N = 107).

Ways to more effectively support weight loss	N (%)
More training on Michigan Medicine weight loss resources and programs	78 (72.9%)
More training on effective dietary counseling	67 (62.6%)
Increased knowledge of effective self-help resources (e.g., mobile health tools, books, websites)	75 (70.1%)
More support from clinic staff (e.g., brief lifestyle counseling by medical assistant)	53 (49.5%)
Peer support programs	47 (43.9%)
Increased on-site access to dietitian	58 (54.2%)
Increased reimbursement for obesity management	56 (52.3%)
Clinical reminders (e.g., Best Practice Alerts)	19 (17.8%)
Clinical decision support tools (e.g., order sets)	38 (35.5%)
Other (please specify)	16 (15.05%)
None of the above	2 (1.9%)

Over one-third of survey respondents reported interest in obtaining certification in obesity medicine through ABOM (n = 41, 39.1%). Two respondents (1.9%) reported being engaged in the process of obtaining ABOM certification.

### Qualitative results

#### Qualitative results

Though we intended to conduct 20 interviews, data saturation—the point at which no new codes emerged from the data—was achieved after 16 interviews [43]. Among 16 interview participants, five (31%) identified as male and seven (44%) specialized in Family Medicine. Additional participant interview characteristics are shown in Table 1.

#### PCPs’ current obesity treatment practice patterns and perceived barriers to obesity treatment

Interview data revealed three key themes relating to PCPs’ obesity treatment practice patterns and barriers. First, PCPs of lack training in obesity medicine constrains use of obesity treatment options. Second, system-level factors including strict program eligibility criteria and lack of insurance coverage for certain programs further limits PCPs’ use of obesity treatments. Third, PCPs tailor obesity treatment discussions to patients’ co-morbidities, preferences, and perceived level of motivation.

*Theme 1*. *PCPs of lack training in obesity medicine constrains use of obesity treatment options*. Interview participants noted the importance of helping patients to manage their weight but acknowledged their own limitations in doing so. One PCP explained, *“I think [weight management is] an overwhelmingly important issue for my patients*, *and I don’t feel like I have enough training to be able to give good evidence-based counseling*, *or initiate treatment… “[I’m] not feeling confident that I’m using the right tools*, *skills*, *medications to…support [patients’ weight loss]*.*”(P2)* PCPs commonly used personal or anecdotal weight management experiences to guide obesity treatment recommendations. For example, one PCP noted, *“I favor MyFitnessPal*, *because I lost a ton of weight on it…I was obese in medical school and training*, *and so I’d use calorie counting to lose a lot of weight and [I am now] actually normal body weight*.*”(P1)* With regards to use of anti-obesity medications, few PCPs reported comfort with prescribing the full range of United States Food and Drug Administration-approved anti-obesity medications [44]. One PCP noted, “*I’m not a huge fan of the drugs…most of them are not covered*. *So*, *I have to warn patients that…it’s just a band aid*. *And*, *and you really just need to make lifestyle changes*.*”(P3)* Rather, PCPs were more comfortable prescribing glucagon-like peptide receptor 1 agonists [GLP1-RAs], which are commonly used to treat patients with type 2 diabetes and increasingly used to treatment patients with obesity. One interview participant noted, *“…I talk to them about…the GLP1 agonists*, *and usually it gets covered because they have diabetes*. *But then I talk to them about…this will help you lose weight*. *Let’s try to get you to the highest dose*.*”(P12)*

*Theme 2*: *System-level factors including strict program eligibility criteria and lack of insurance coverage for certain obesity treatment options further limits PCPs’ use of obesity treatments*. PCPs perceived that certain health system obesity treatment resources have limited capacity and narrow program eligibility criteria, including specific BMI, co-morbidity or insurance requirements. For example, when discussing the health systems’ weight management treatment options (e.g., meal replacement program; Mediterranean-style diet and physical activity program), one PCP stated, *“[I]t seems like the inclusion and exclusion criteria…are so narrow that few of my patients can do them…I’ve given lots of referrals [for the meal replacement program]*.*”(P8)* PCPs also reported limited insurance coverage and high out-of-pocket costs for anti-obesity medications. *“Oftentimes we’ll talk [weight management medications]*. *It’s just that the…copay is so high…[and]…the insurance won’t approve it*.*” (P14)* As a result of system-level barriers, PCPs commonly refer patients for nutrition counseling with dietitians and/or recommend community-based lifestyle change programs (e.g., Diabetes Prevention Program, Weight Watchers), although they acknowledged variable effectiveness of these treatment options. With regards to nutrition counseling, one PCP noted, *“I happen to have a very low socioeconomic population*, *so a lot of my patients are limited on food [budget]*, *a lot of them use food pantries*, *and it just really limits what you can do…Some nutritionists talk to patients about all these healthy things that my patients can’t afford*.*”(P1)*

*Theme 3*. *PCPs tailor obesity treatment discussions to patients’ co-morbidities*, *preferences*, *and perceived level of motivation*. PCPs do not routinely discuss weight management with patients, but rather wait for specific clinical opportunities (e.g., health maintenance examinations) or patient-initiated conversation. One PCP voiced concern that routine discussion of body weight may offend patients: “*I can bring up [weight status] with knee pain*. *But if it’s something…[not a condition] easily relatable [to body weight] or the patient doesn’t bring it up themselves*, *or it’s not a health maintenance exam*, *it’s harder for me to… find a way to bring it in without feeling like I’m being judgmental*. *So typically*, *in those situations I won’t say anything [about weight]*.*”(P7)* PCPs reported that referrals to health system obesity treatment resources was often guided by patients’ specific requests. One PCP explained that “*the people who [I refer to bariatric surgery] tend to be the…people who are already thinking about it*.*”(P12)* Additionally, PCPs tailored the frequency of weight management follow-up to perceived levels of patient motivation. One PCP noted, *“[For most patients who aren’t] extremely motivated*, *[follow-up] would just be the once-a-year physical*, *plus…and if I refer to the dietician*, *the visits with them*.*”(P13)*

#### PCPs’ perceived opportunities to improve obesity treatment

Interview data revealed three key themes relating to PCPs’ perceived opportunities to improve obesity treatment. First, PCPs desired clinic-based resources to support evidence-based obesity treatment. Second, PCPs desired multidisciplinary care teams to support obesity treatment initiation and follow-up. Third, PCPs desired additional training in obesity medicine.

*Theme 1*: *PCPs desired clinic-based resources to support evidence-based obesity treatment*. PCPs requested better education to help them deliver evidence-based obesity treatment, refer patients to appropriate health system programs, and determine coverage information for anti-obesity medications: *“If there was just a list or all the possible [programs]*. *If you need weight loss*, *here’s a list of all the programs that are available*. *So that was something we could give to patients*.*”(P10)* Another PCP explained: *“[A] lot of my use of [anti-obesity medications]has been from me learning about it on my own*. *So if there’s* …*any sort of repository of…articles or something I could go to as…a localized resource to learn more about them*, *I think that would be super helpful*.*”(P5)*

*Theme 2*: *PCPs desired multidisciplinary care teams to support obesity treatment initiation and follow-up*. PCPs desired an inter-disciplinary, primary care-based team including an obesity medicine specialist, nutritionist, and care manager to assist them in treating patients with obesity. As one PCP noted, *“[Such an approach would be] more intensive than just seeing your PCP*, *but less intensive than the…[meal replacement] weight management program that has so many restrictions associated with it that make it…not accessible for many people*.*”(P12)* PCPs also raised concerns about the workload of insurance prior authorizations for anti-obesity medications and desired a team or support to address the authorization process. One PCP felt comfortable providing evidence-based obesity treatment but acknowledged the challenge of providing intensive lifestyle counseling: *“What I need is actually an adjunctive [team]member who actually specializes in…psychology*, *and like…sort of mental health and addiction and motivational interviewing to help*.*” (P16)* Another PCP requested the support of a team member who had familiarity with community-based obesity treatment options to enhance the delivery of preference-sensitive obesity treatment: *“It’s almost like a social worker*. *It would have to be definitely community-based outreach and*, *and culturally appropriate*, *and probably almost neighborhood specific*.” *(P10)*

*Theme 3*: *Interest in obtaining certification in obesity medicine through ABOM*. Some PCPs reported the desire for formal training in obesity medicine, though acknowledged lack of time and inadequate financial support for ABOM certification as a key barrier. One PCP stated, *“I’ve actually been weighing [board certification] for a while…[obesity is] just such an important health problem*, *that…I think this makes a lot of sense for all of us to do…I would be very interested in doing it*.*”(P12)* Another PCP similarly expressed interest in ABOM certification but noted, *“[obtaining certification is] thousands of dollars”* and pursuing the option would depend on “*if I magically had…extra hours in the day and [the] university was gonna pay for it*.*”(P13)* One interview participant with ABOM certification commented on the benefits of training: “*Before I passed the exam*, *I felt like I was faking it*. *But now… I’ve been making it a point to [practice obesity medicine] and to talk to patients about obesity so much*, *that I really feel comfortable doing it*.*”(P1)*

#### Integrated results

As shown in **Table 3,** survey respondents desired increased access to resources to support delivery of evidence-based obesity treatment. Qualitative interview data were concordant with these findings, with interview participants expressing specific desire for a clinical tool or algorithm to guide selection of obesity treatment options based on individual patients’ BMI, co-morbidities, and insurance coverage. Survey respondents expressed need for enhanced team-based obesity treatment, including lifestyle counseling by non-PCP team members and increased access to on-site dietitians. Interview participants specifically suggested (1) lifestyle counseling by team members such as medical assistants or nurses, (2) assistance with anti-obesity medication prescribing from clinical pharmacists, and (3) staff support for completing insurance prior authorizations for anti-obesity medications. Nearly half (44%) of survey respondents felt that integration of an ABOM Diplomate into primary care teams could improve obesity treatment. While most interview participants agreed that access to an ABOM Diplomate could improve obesity treatment, a minority expressed concern that this could fragment care or duplicate PCPs’ efforts. Over one-third of survey respondents (39.1%) indicated interest in obtaining ABOM certification. Interview participants similarly expressed interest in ABOM certification, though noted potential barriers to training, including existing workload burden, lack of time, and relatively high training and examination costs.

## Discussion

This mixed methods study aimed to identify PCPs’ perceived opportunities to improve obesity treatment in primary care settings. To contextualize these opportunities, we first explored PCPs’ perceived barriers to obesity treatment.

Consistent with prior literature [15, 45, 46], key barriers to obesity treatment identified by study participants included lack of training in obesity medicine, short visit durations with competing clinical demands, and patients’ lack of insurance coverage or high out-of-pocket costs for certain treatment options (e.g., meal replacement program, anti-obesity medications).

The majority (92.5%) of survey participants reported only using clinical judgement—rather than clinical practice guidelines—when treating patients with obesity. This is consistent with prior work suggesting that PCPs may not fully recognize inconsistencies between their own obesity treatment practice patterns and guideline recommendations. Specifically, among a nationally representative cohort of health professionals, providers’ understanding of behavior counseling and anti-obesity medication prescribing recommendations were inconsistent with evidence-based guidelines [47]. In contrast, obesity treatment provided by ABOM Diplomates demonstrates 30%-65% concordance with obesity treatment guidelines recommendations [48].

Study participants acknowledged that effective obesity treatment requires team-based, collaborative care and recognized that medical assistants, nurses, and pharmacists may play key roles in supporting obesity care delivery. This is consistent with prior calls for a multidisciplinary approach to obesity management [49, 50] with integration of non-PCP team members (e.g., nurses) [51] as well as clinical and community weight loss resources [52, 53]. Other health systems have utilized Electronic Health Record-based clinical decision support tools to guided PCPs’ obesity treatment decision-making [53]. While some PCPs in our study expressed interest in clinical decision support tools, the real-world acceptability of such approaches may be limited, as even guided treatment options require time for discussion. Nearly 40% of survey participants and 70% of interview participants indicated the desire for the integration of an obesity medicine expert (e.g., ABOM Diplomate) within primary care teams to provide a comprehensive obesity assessment with personalized treatment plan recommendations.

To our knowledge, this is one of the first studies to explore PCPs’ interest in obtaining ABOM certification. Forty percent of survey participants expressed interest in training and certifying in obesity medicine through ABOM, with two participants in the process at the time of survey completion. Despite relatively high general interest in ABOM training and certification, interview participants expressed reluctance due to the costs of training and board examination and a lack of protected time to complete 60 Continuing Medical Education credits within 36 months [54].

To overcome barriers and capitalize on opportunities indicated by PCPs in this study, we are developing and testing a Weight Navigation Program (WNP) [55]. The WNP aims to integrate an ABOM Diplomate into primary care teams to serve as an obesity medicine consultant and enhance the delivery of personalized, effective obesity treatment through use of health system, community, and pharmacotherapeutic weight management resources. ABOM Diplomates who currently provide care within the Weight Navigation Program are Family Medicine physicians who devote approximately 4 hours per week to this program.

### Limitations

This study has several key limitations. First, the study was conducted in a single academic health system, and the findings may not be generalizable to other treatment settings. Second, given the voluntary nature of both the survey and interview, the results may be subject to respondent bias, as PCPs interested in this topic may have been more likely to participate and their responses may not reflect those of PCPs with less interest in or experience with weight management. Third, we evaluated PCPs’ self-reported practice patterns. Due to social desirability bias, this may have resulted in an overestimation of PCPs’ actual practice patterns. Our findings, however, are consistent with prior literature and reveal substantial gaps in the delivery of evidence-based weight management treatment. Fourth, our survey response rate was relatively low (30.6%), which may have been due to the concomitant burden of the COVID-19 pandemic during the study period.

## Conclusions

PCPs in a large, academic health system face barriers to providing evidence-based obesity treatment, which will be difficult—if not impossible—to overcome without health system innovations and health policy changes. Health systems looking to improve their ability to provide effective, evidence-based obesity treatment may identify PCPs with specific interests in obesity medicine and support their ABOM certification by reimbursing training costs and reducing clinical effort to allow for training and board examination. Moreover, ABOM-certified PCPs must be allowed clinical effort to specifically devote specifically to obesity medicine. Health systems may further enhance patients’ access to effective obesity treatment by allocating sufficient resources to support and expand the full range of obesity treatment options, including low-cost options commonly utilized by PCPs (e.g., nutrition services). Lastly, the potential population health benefits of obesity treatment may only be realized when all patients have access to effective obesity treatments. This will require enhanced reimbursement for obesity treatment and insurance coverage for the full range of treatment options, including comprehensive lifestyle intervention, anti-obesity medications, medical weight management programs, and bariatric surgery.

## Supporting information

S1 Appendix Summary of survey items, item numbers, response options, and sources. Item numbers indicate order in baseline survey.
(DOCX)

S2 Appendix Physician interview guide. (DOCX)

S1 Data Minimal anonymized data set. (XLSX)
