# Supplementary material for: Primary care providers’ perceived barriers to obesity treatment and opportunities for improvement: A mixed methods study
Source: PLoS One. 2023 Apr 18;18(4):e0284474. doi: 10.1371/journal.pone.0284474 (PMC10112804; doi:10.1371/journal.pone.0284474)
Supplement: S1 Appendix — Item numbers indicate order in baseline survey. (DOCX) [file pone.0284474.s001.docx]

| **Appendix 1.** Summary of survey items, item numbers, response options, and sources. Item numbers indicate order in baseline survey. | | |
| --- | --- | --- |
| *Introduction. The purpose of this survey is to: (1) understand barriers to obesity management in Michigan Medicine’s primary care clinics; and (2) identify opportunities for improvement. We appreciate your input and understand your time is valuable. Your answers will remain anonymous.* | | |
| **Question** | **Response options** | **Origin of question items** |
| **Obesity treatment experience and knowledge** | | |
| *Please use the sliding scale below to estimate:* | | |
| The percent of patients that you care for who have obesity. | Answer choices on a sliding scale from 0 to 100. | Developed by study team and iteratively revised based on feedback from colleagues and weight management treatment experts. |
| The percent of clinic encounters in which you discuss weight management with your patients with obesity. |  |  |
| *Assessment of perceived clinically significant weight loss threshold* | | |
| In your opinion, how much weight does a patient with obesity need to lose to start to improve his or her health? | - 5% - 10% - 15% - 20% - It depends on the patient's starting weight - I don't know - Other (please specify) | Developed by study team and iteratively revised based on feedback from colleagues and weight management treatment experts. |
| **Current obesity treatment practice patterns and barriers** | | |
| *Over the past year, how often have you used the following treatments or made referrals to the following programs when treating patients with obesity?* | | |
| Dietitian | - Very often (Weekly) - Often (A few times a month) - Not very often (a few times a year) - I do not use this. | Developed by study team and iteratively revised based on feedback from colleagues and weight management treatment experts. |
| Anti-obesity/weight control medication |  |  |
| Lifestyle change program (e.g., Weight Watchers, Diabetes Prevention Program, MHealthy) |  |  |
| Hunger Within Program |  |  |
| Metabolic Fitness Program (through Preventive Cardiology) |  |  |
| Endoscopic bariatric weight loss program (balloon device; aspiration device; endoscopic sleeve gastroplasty) |  |  |
| Weight loss surgery (i.e. bariatric surgery) |  |  |
| Weight management program (through Metabolism, Endocrinology, and Diabetes) |  |  |
| Self-help resources |  |  |
| *If I do not discuss weight management with a patient with obesity, it is because:* | | |
| I already know that losing weight is not a priority or goal for the patient. | - Strongly Agree - Agree - Neither Agree nor Disagree - Disagree - Strongly Disagree | Adapted from:  Croghan IT, Ebbert JO, Njeru JW, et al. Identifying Opportunities for Advancing Weight Management in Primary Care. *J Prim Care Community Health.* 2019;10:2150132719870879. doi:10.1177/2150132719870879  Sharma AM, Bélanger A, Carson V, et al. Perceptions of barriers to effective obesity management in Canada: Results from the ACTION study. *Clin Obes.* 2019;9(5):e12329. doi:10.1111/cob.12329 |
| The patient has other health concerns or conditions that are more urgent. |  |  |
| The patient does not have any weight-related health problems. |  |  |
| The patient does not tell me he/she/they want(s) to lose weight. |  |  |
| The patient already knows what he/she/they need(s) to do to manage their weight. |  |  |
| I don't want to offend my patient by discussing his/her/their weight. |  |  |
| It is not my role as a physician to counsel on weight loss. |  |  |
| I am not confident in my ability to make specific weight loss/control recommendations. |  |  |
| I do not believe that the patient will take the necessary steps to lose weight. |  |  |
| Helping patients to lose weight is not a priority for me. |  |  |
| The visit duration is too short. |  |  |
| The available weight loss strategies are not effective. |  |  |
| The patient does not have insurance coverage for the available weight loss resources/programs. |  |  |
| I cannot bill for conversations about weight loss. |  |  |
| *Select all that apply to the following question:* | | |
| Which, if any, of the following obesity management resources do you use when treating patients with obesity? (Select all that apply) | - ACCE/ACE Algorithm for the Medical Care of Patients with Obesity - Pharmacologic Management of Obesity: An Endocrine Society Clinical Practice Guidelines - 2013 AHA/ACC/TOS Guidelines for the Management of Overweight and Obesity in Adults - Obesity Medicine Association Obesity Algorithm - I use my clinical judgement - Other (please specify) | Adapted from 2019 American Board of Obesity Medicine Diplomate Survey |
| **Opportunities to Improve Obesity Treatment in Primary Care Settings** | | |
| *Please consider the primary care clinic where you work when answering the following questions:* | | |
| My primary care colleagues consider obesity treatment to be a priority. | - Strongly agree - Agree - Neither agree nor disagree - Disagree - Strongly disagree | Adapted from:  Ehrhart et al, Ehrhart, M.G., Torres, E.M., Hwang, J. et al. Validation of the Implementation Climate Scale (ICS) in substance use disorder treatment organizations. Subst Abuse Treat Prev Policy 14, 35 (2019). <https://doi.org/10.1186/s13011-019-0222-5> |
| My clinic's leadership considers obesity treatment to be a priority. |  |  |
| Michigan Medicine offers adequate weight management resources. |  |  |
| *Now we would like you to think of ways you can improve the lives of patients with obesity.* | | |
| Which of the following would better help you support weight loss for your patients (select all the apply): | - More training on Michigan Medicine weight loss resources and programs - More training on effective dietary counseling - Increased knowledge of effective self-help resources (e.g. mobile health tools, books, websites) - More support from clinic staff (e.g. brief lifestyle counseling by medical assistant) - Peer support programs - Increased on-site access to dietitian - Increased reimbursement for obesity management - Clinical reminders (e.g. Best Practice Alerts) - Clinical decision support tools (e.g. order sets) - Other (please specify) - None of the above | Developed by study team and iteratively revised based on feedback from colleagues and weight management treatment experts. |
| *Obesity medicine is an emerging field. The American Board of Obesity Medicine (ABOM) maintains standards for assessment and credentialing physicians. Physicians can receive ABOM-certification by completing CME requirements and passing a certifying examination.* | | |
| I am certified in obesity management through the American Board of Obesity Medicine | - Yes - No | Developed by study team and iteratively revised based on feedback from colleagues and weight management treatment experts. |
| *If “yes” to “I am certified in obesity management through the American Board of Obesity Medicine”* | | |
| Please indicate how many years you have been certified: | Open-ended | Adapted from 2019 American Board of Obesity Medicine Diplomate Survey |
| How many hours per week do you devote to obesity management: | Open-ended |  |
| *If “no” to “I am certified in obesity management through the American Board of Obesity Medicine”* | | |
| Please indicate your interest in becoming certified: | - I am not interested in becoming certified. - I am in the process of becoming certified. - I am interested in becoming certified but I have not started the process. - I am unsure. - Other (please specify) | Developed by study team and iteratively revised based on feedback from colleagues and weight management treatment experts. |
| **Primary Care Provider Characteristics** | | |
| *Tell us a bit about yourself.* | | |
| What is your gender? | - Male - Female - Other (please specify) - Prefer not to answer | Developed by study team and iteratively revised based on feedback from colleagues and weight management treatment experts. |
| What is your specialty? | - Family Medicine - Internal Medicine - Med-Peds - Other (please specify) |  |
| I have received special training in obesity management. | - Yes (please specify where you received the training) - No |  |
| How many years have you been practicing care (not including residency training)? | - Less than 5 years - Between 5 and 10 years - More than 10 years |  |
| How many half days per week do you devote to outpatient clinical practice? | - 1-2 - 3-4 - 5-6 - 7-8 - Other (please specify) |  |
| Can we contact you directly to participate in a brief interview regarding obesity management in primary care? | - Yes - No |  |
| Is there anything else you would like to tell us? | Open-ended |  |
