# Supplementary material for: Primary care providers’ perceived barriers to obesity treatment and opportunities for improvement: A mixed methods study
Source: PLoS One. 2023 Apr 18;18(4):e0284474. doi: 10.1371/journal.pone.0284474 (PMC10112804; doi:10.1371/journal.pone.0284474)
Supplement: S2 Appendix — (DOCX) [file pone.0284474.s002.docx]

**Physician Interview Guide**

**Introduce self**

Hello, my name is [name of interviewer]

**Discuss purpose of the interview group**

We are interested in understanding your experiences with treating patients with obesity in your primary care practice. We want to understand the challenges you face and also the opportunities you see to improve treatment for patients with obesity in your clinic and our health system. We would also like to gather your thoughts and feedback on a specific program that we are developing to support weight loss among primary care patients with obesity.

I want to encourage you to answer honestly and share any thoughts you may have. There is no right or wrong answer, and you will not be penalized for anything you say. We care about your opinion, so that we can make an even better program that could benefit others.

You will receive a $40 gift card for participating in this interview.

**Describe how we will assure confidentiality and answer any questions.**

I want to take a minute to tell you what happens with the information you provide for us today. I would like to record this conversation so that we don’t miss any of the comments that you share. People working on this study will be the only ones who will use the interview recordings. The findings from this interview will be de-identified and reported in aggregate with other interviews.

As a reminder, you are not obligated to answer any question that makes you feel uncomfortable and you are not required to participate. You may leave the interview at any time.

I’m going to turn on the recorder now and we will get started. ***Turn on recorder***

The rest of the conversation is being recorded for research purposes

To proceed with this interview and to allow us to use the information, we need to confirm that you agree to participate. Do you consent to participate in this study?

If “yes”: Great, let’s get started.

**Interview**

***First, we’d like to understand how and when you discuss patients’ weight and the factors the influence your decision to discuss weight.***

1. Tell me about your current process identifying patients who might benefit from weight loss.
2. Tell me about the factors that might make you **more likely** to bring up the topic of weight loss during a visit with a patient with overweight or obesity.

**Probe, if needed:**

- Are there patient characteristics or obesity-related co-morbidities that might make you more likely to discuss weight loss?
- Are there certain visit types or chief complaints where you are more likely to discuss weight loss?

***Next, I’d like to understand what you are already doing to treat patients with overweight or obesity.***

1. Tell me about how you help patients with overweight or obesity to set a weight loss goal, if at all.
2. When you are managing weight loss, how frequently do you see patients for follow-up appointments, if at all?
3. Based on your prior survey responses, it looks like you commonly use [NAME PROGRAM(S) or RESOURCE(S)] to help patients lose weight. Is this correct? Are there other programs or resources that you use?
   1. What are your experiences with these resources?
   2. How do these resources work for your patients?
   3. Any particularly positive or negative experiences?
4. **If provider does not discuss self-help resources in question above:** Tell me about any self-help resources that you recommend to help patients lose weight, such as phone apps for calorie tracking or fitness tracking.
   1. What are your experiences with these resources?
   2. How do these resources work for your patients?
   3. Any particularly positive or negative experiences?
5. Tell me about any experiences you have had referring patients to a nutritionist to develop a meal plan for weight loss.
   1. What have your patients told you about working a nutritionist?
   2. What are your experiences with nutritionist referrals for weight loss? Any particularly positive or negative experiences?

***We are interested in knowing your thoughts about how the health system (Michigan Medicine), your primary care practice, or your colleagues and staff could better support you in caring for patients with obesity.***

1. If you could wave a magic wand, what two or three things could Michigan Medicine do that would help support you in caring for patients with obesity.
2. Tell me about any ideas you might have for how your specific practice could better support you in caring for patients with obesity.

Probe, if needed:

- Do you have any colleagues with obesity medicine expertise?
- Who do you turn to, if anyone at all, if you have specific questions about how to manage a patient with obesity?
- Are there clinical staff members who could support you in caring for patients with obesity? What support could they provide?

***I’d like to get your thoughts about the Weight Navigation Program (WNP) that our team is developing to better support PCPs and their patients with obesity. This program would embed a board-certified obesity medicine primary care physician at your clinic site. Patients referred by their PCP would meet with the obesity medicine specialist—known as the “Weight Navigator” –to develop a personalized weight loss treatment plan that uses Michigan Medicine, community, and self-help resources. The Weight Navigator might also make recommendations for the PCP to change medications that can prevent weight loss (for example, insulin), or add medications to support weight loss. The program would include self-monitoring tools and a population health manager to make sure patients are achieving their goals. The patient would follow-up with PCPs during regularly scheduled visits, and the PCP would use the Weight Navigator’s plan to guide additional weight management decisions, if needed. This might include initiating referrals or starting weight loss medications. The Weight Navigator would be available for follow-up questions, and follow-up appointments with the Weight Navigator could be scheduled, if needed.***

1. Based on this information, do you have any questions about the program?
2. How helpful would the WNP model be for you and your patients with obesity?
3. Would you consider referring patients to this type of program?
4. Are there any problems you see with this type of program?
5. What suggestions do you have to make a program like this work better for you and your patients?
6. The goal of the WNP would be to help patients engage in a preference-sensitive weight loss program or treatment. However, the Weight Navigator will clearly outline in the consult note additional treatment options that could be considered if the patient does not meet weight loss goals with the first approach. Would you feel comfortable assessing patient progress and implementing additional interventions detailed in the WNP consult note, if needed?
   1. Are there certain referrals you would not feel comfortable placing?
   2. Are there certain lab tests you would not feel comfortable placing and/or interpreting?
   3. What additional suggestions do you have, if any, to make this program work better for you and your patients?

**Different physicians have different practice styles and levels of comfort with obesity medicine. The next questions ask about your current practice patterns and how the WNP could complement or add value to your current practice.**

1. Tell me about how you counsel patients, if at all, about popular dietary strategies such as low-carb, keto, plant-based or intermittent fasting?
   1. Think about a scenario in which the patient has self-initiated a strategy like keto or intermittent fasting and is losing weight. Does this change your counseling strategy for any of these popular dietary strategies?
   2. Tell me about any concerns you would have if an obesity specialist recommended low-carb, keto, plant-based or intermittent fasting to your patients?
2. Tell me about how you use weight loss medications to treat patients with obesity, if at all.
   1. [If physician **does not use** weight loss medications, ask] Tell me about the reasons you do not prescribed weight loss medications.
   2. [If physician **does use** weight loss medications, ask] Thinking of the patients you are treating, how do you decide to use a weight loss medication? Which medications do you most commonly use? What has your experience been?
   3. Consider a scenario in which the Weight Navigator provides clear recommendations on how to initiate, monitor, and titrate weight loss medications via a MiChart consult note. Would you be comfortable implementing such recommendations?
   4. Tell me about any training, tools, or resources that might support your use of weight loss medications.
3. In a prior survey response, you indicated that you [were / were not] interested in becoming certified in obesity medicine through the American Board of Obesity Medicine. As you may recall, certification requires 60 CMEs and passing a standardized examination.

**Probe:**

- 1. Tell me more about why you [are/are not] interested in obesity medicine certification.
  2. For interested providers:
     1. What support do you need to obtain certification?

1. Is there anything else you would like us to know about this topic?

**Conclusion**

Thank you very much for your time and participation. We will send you your $40 gift card in the mail. Could you please provide your preferred address?

****Turn off recorder****
